# Role of Regulatory and Proinflammatory T-Cell Populations in Allergic Diseases

**DOI:** 10.1097/wox.0b013e3181629ae3

**Published:** 2008-01-25

**Authors:** Kanami Orihara, Susumu Nakae, Ruby Pawankar, Hirohisa Saito

**Affiliations:** 1 Department of Allergy and Immunology, National Research Institute for Child Health and Development, 2-10-1 Okura, Setagaya-ku 157-8535, Tokyo, Japan; 2 The Atopy Research Center, Juntendo University; 3 The Department of Otolaryngology, Division of Rhinology and Allergy, Nippon Medical School, Tokyo, Japan

**Keywords:** helper T cells, regulatory T cells, interleukin 17, mast cells, thymic stromal lymphopoietin

## Abstract

Regulatory T (T_reg_) cells are considered to inhibit the development of both type 1 (T_h_1) and type 2 helper T (T_h_2) cells. However, it is recently reported that there are reduced numbers of T_reg _cells in patients with allergic diseases as compared with individuals who have high levels of serum immunoglobulin E and blood eosinophils but are asymptomatic. Therefore, T_reg _cells may suppress the onset of allergic disease by down-regulating other types of immune cells besides T_h_1 and T_h_2 cells. The newly discovered interleukin 17-producing helper T cells that are responsible for autoimmune inflammatory diseases may counteract T_reg _cells even in allergic diseases. The T_h_2 cells that are capable of producing of high levels of tumor necrosis factor-*α *may also be involved in inflammation in allergic diseases. In this review, we further discuss the role of T_h_1, T_h_2, interleukin 17-producing helper T cells, and T_reg _cells in allergic diseases by using the balancing square model and the factors differentiating between patients with clinical manifestations of allergic symptomatic and atopic individuals who are sensitized but asymptomatic.

## T_h_1/T_h_2 paradigm theorizing for allergic diseases is on the rise

There is an inverse correlation between the levels of endotoxin in house dust and the incidence of atopic sensitization and hay fever [1]. The major role of endotoxin is considered to be the stimulation of macrophages/antigen-presenting cells to produce interleukin 12 (IL-12), which triggers the development of antigen-specific type 1 helper T (T_h_1) cells and inhibits the development of type 2 helper T (T_h_2) cells. As such, the hygiene hypothesis associated with an increasing prevalence of allergic diseases has been theorized by the T_h_1/T_h_2 paradigm [2] since a long time.

**Figure 1:**
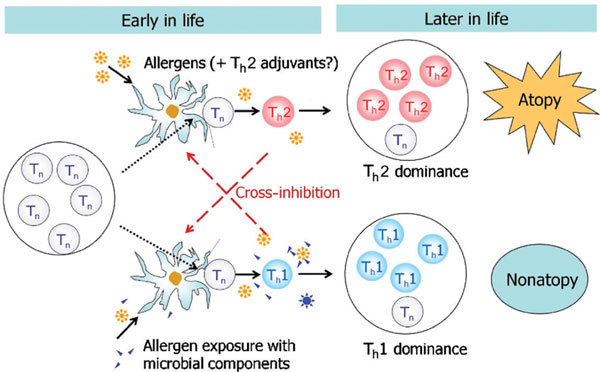
**Environment surrounded by microbial components is effective only during early childhood**. Only T_n_'s are present during infancy. The T_h_2-dominant immune system will develop when the immune system is exposed to allergens without microbial components such as endotoxin. Allergen-associated T_h_2 adjuvants such as prostaglandins may enhance T_h_2 development. On the other hand, antigen/allergen-specific T_h_1 cells can develop by simultaneous exposure to antigen/allergen and microbial components. After childhood, microbial exposure cannot drastically alter the proportion of T_h_1/T_h_2 balance because the proportion of T_n _is decreased. T_n _indicates naive helper T cells.

Most epidemiological studies supporting the hygiene hypothesis also indicate that the preventive effect on allergy of an "unhygienic" environment surrounded by many microbial components is limited to early childhood [1]. According to the classic T_h_1/T_h_2 paradigm theory, this could be typically speculated as shown in Figure 1. A T_h_2-dominant immune system develops in an individual when the immune system is exposed to allergens without prior exposure to microbial components such as endotoxin early in life. On the other hand, the development of allergen (antigen)-specific T_h_1 cells is triggered by simultaneous exposure to the antigen and microbial components. After childhood, the proportion of T_h_1/T_h_2 cells is not drastically altered by microbial exposure because of a decrease in the number of naive helper T cells that can react with common allergens.

The incidence of T_h_1-mediated autoimmune diseases is known to have increased in the last half century in parallel with the increase of T_h_2-mediated allergic diseases [3]. The classic T_h_1/T_h_2 paradigm cannot be used to explain this point.

## Roles of various regulatory T-cell populations

Several subsets of CD4^+ ^cells are able to prevent immune responses against self-antigens or allergens. These cells are called regulatory T (T_reg_) cells. Because T_reg _cells inhibit both T_h_1- and T_h_2-cell development in vitro, increases in the incidence of T_h_1 diseases and T_h_2-mediated diseases are now thought to be related to an insufficient development of T_reg _cells. However, there is no evidence yet whether the "hygienic" environment with exposure to less microbial components during early life affects the development of T_reg _cells.

Among the several subsets, naturally occurring T_reg _(nT_reg_) cells have been well investigated. These cells originate in the thymus, express the repertoire of CD4^+^CD25^+ ^(mouse) or CD4^+^CD25^high ^(human) and the transcription factor forkhead box protein P3 (Foxp3), and have a major role in modulating the activity of self-reactive cells by inducing the destruction of autoreactive T cells mainly via cell-to-cell contact-dependent mechanisms [4]. Therefore, nT_reg _cells are considered to mainly have a preventive role in autoimmune diseases.

In humans, a population of CD4^+^CD25^high ^T cells with regulatory function very similar to nT_reg _cells but derived from peripheral memory CD4^+^CD25^- ^T cells has recently been described [5]. They are called adaptive T_reg _(aT_reg_) cells [6] or inducible T_reg _(iT_reg_) cells. One of iT_reg_-cell types is the IL-10-producing type 1 T_reg _(Tr1) cells, whose suppressive function has been well documented in allergy and autoimmunity [7]. The term "iT_reg_" is often used for the IL-10-producing Tr1, whereas the term aT_reg _is often used as CD4^+^CD25^high ^T cells derived from peripheral memory CD4^+^CD25^- ^T cells. Therefore, in this review, we have used the term aT_reg _as Foxp3^+ ^CD4^+^CD25^high ^T cells as shown in the recent review article written by Bacchetta et al [8]. Nevertheless, all subsets of T_reg _cells require a cytokine, transforming growth factor-β (TGF-β), for their development. The IL-10 is also produced not only by Tr1 cells, but also by various cell types including regulatory dendritic cells that can induce aT_reg. _[9] As such, these 2 cytokines play an important role in immune regulation.

Forkhead box protein P3 mutant mice develop an intense multiorgan inflammatory response, including allergic airway inflammation, striking hyperimmunoglobulinemia E, eosinophilia, and dysregulated T_h_1 and T_h_2 cytokine production [10,11]. In humans, genetic defects in Foxp3 cause immune dysregulation, polyendocrinopathy, and enteropathy, X-linked (IPEX) syndrome [12]. Most IPEX patients have food allergy and atopic dermatitis-related symptoms immediately after birth. It is thus suggested that Foxp3^+ ^T_reg _cells play an important role in regulating common allergic disorders and IPEX. The number of Foxp3^+ ^T_reg _cells is decreased in skin lesions in patients with atopic dermatitis and in patients with psoriasis [13].

Although Foxp3 is transiently expressed by antigen-activated helper T cells, [14] only persistent and high-level Foxp3 expression is related to the immunosuppressive functions.

## IL-17-producing helper T cells that triggered the major revision for T_h_1/T_h_2 theory

A major role for the cytokine IL-17 has recently been described in various murine models of immune-mediated tissue injury, organ-specific autoimmunity, allergic disorders, and microbial infections. Interestingly, interferon-γ (IFN-γ) derived from T_h_1 cells often prevents the IL-17-mediated inflammation in mice with experimental autoimmune diseases [15,16]. A T-cell subpopulation that exclusively produces IL-17 (T_h_17) is now credited for causing and sustaining tissue damage. Human and mouse T_h_17 cells may require different sets of cytokines for their development. Thus, the identification of the T_h_17 cells triggered a shift in the immunologists' perspectives regarding the basis of tissue damage or auto-immune diseases, where for more than 20 years the role of T_h_1 cells was considered paramount [16].

However, it has been recently revealed that the expression of IL-17 in human CD4^+ ^T cells may be completely different from that in mice [17]. Unlike mouse, human IL-6 and IL-21 do not induce IL-17 expression in either naive or effector T cells. The TGF-β inhibits human T_h_17-cell development but promotes mouse T_h_17-cell development when costimulated with IL-6 [17]. It should also be noted that in human adult peripheral blood, a large proportion of helper T cells can produce both IFN-γ and IL-4. The proportion of T_h_17 cells in peripheral blood CD4^+ ^cells are consistently less than 1% in the peripheral blood of healthy individuals, and slightly higher among CD4^+ ^T cells derived from patients with Crohn's disease [18]. Autologous T_reg_-cell clones suppress T_h_1 or T_h_2 cells, but not T_h_17 cells [18]. Nevertheless, IL-6 inhibits the development of both human and mouse T_reg _cells [19].

The key cytokine of T_h_17 cells, IL-17, is known to induce the production of proinflammatory cytokines such as tumor necrosis factor-α (TNF-α), IL-1β, IL-6, and proinflammatory chemokines CXCL1, 2, and 8 by acting on various cell types [20]. In humans, sputum IL-17A messenger RNA levels are significantly elevated in patients with asthma as compared with healthy controls [21]. Endogenous IL-17 contributes to the development of allergen-induced airway hyperresponsiveness, and there is also evidence that IL-17 stimulates the release of several cytokines with known capacity for airway remodeling from cells normally residing in the airways [22].

**Figure 2:**
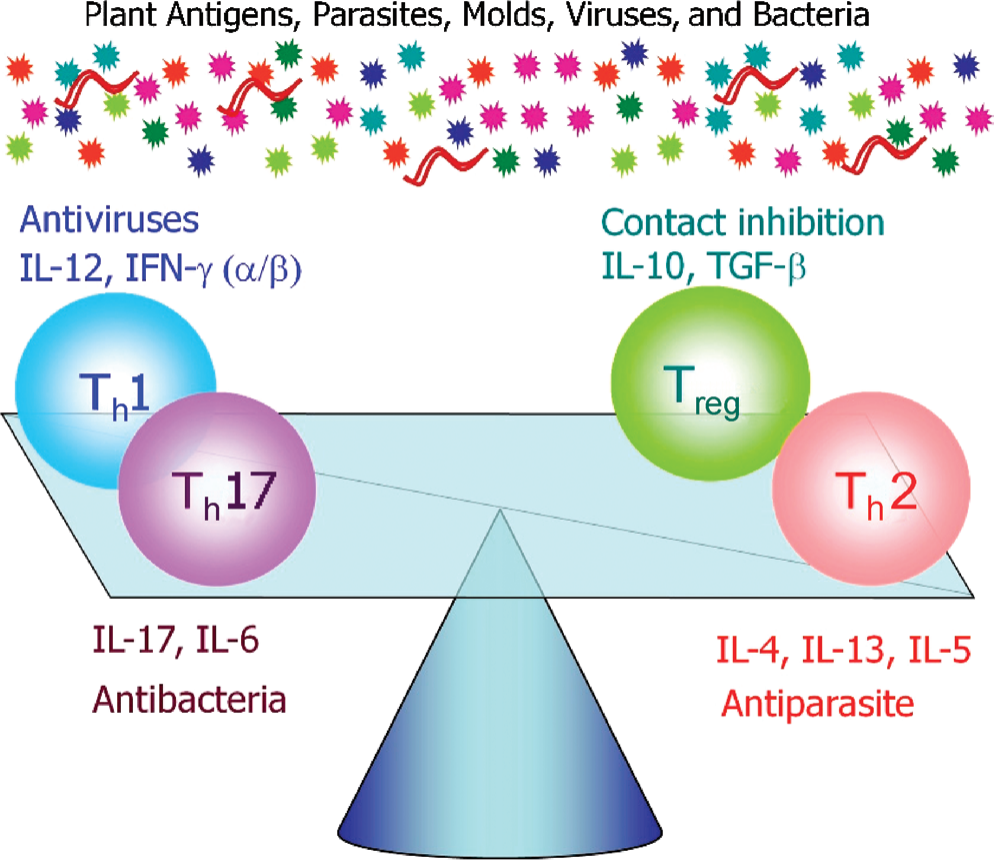
**The balancing square model**. The 4 T-cell types (T_h_1, T_h_2, T_h_17, and T_reg_) antagonize each other. The T_h_1-promoting cytokine, IL-12, is inhibitory to T_h_2-cell development, whereas the T_h_2-promoting cytokine, IL-4, blocks T_h_1-cell development. The T_h_1-derived IFN-γ blocks T_h_17-cell development. The T_reg _inhibits the development of both Th1 and Th2 cells by direct contact.

With the discovery of T_h_17 and T_reg _populations, the balancing square model is now needed to explain the pathogenesis of various immunological diseases. It also enables us to explain the epidemiological data demonstrating an increase in allergic diseases (Figure 2). According to this model, we speculate that substantial amounts of plant antigens, parasites, molds, viruses, and bacteria are required for balancing the total immune system (Figure 2). Autoimmune diseases are now considered to be initiated by an up-regulation of T_h_17 cells and a defect in nT_reg _cell function, whereas peritumor tissues are strikingly infiltrated with Foxp3^+ ^nT_reg _cells implying that these cells impinge upon immunemediated rejection of the tumor [23].

## Role of aT_reg _cells in the onset of allergic diseases

Allergic diseases are caused by uncontrolled T_h_2-based immune responses to environmental antigens. It has been demonstrated that healthy nonatopic subjects have detectable IL-10-producing allergen-specific Tr1-like T_reg _cells, whereas the proportion of these T_reg _cells are very low in symptomatic allergic patients [24].

Several studies suggest a possible defect and impaired function of nT_reg _cells in the pathogenesis of immune responses toward allergens. However, like studies on auto-immune diseases, it is often difficult to distinguish the overlapping phenotypic characteristics between T_reg _cells and activated helper T cells [8].

We recently demonstrated that symptomatic atopic patients had a lower Foxp3^+^CD4^+ ^ratio than asymptomatic controls having similar levels of serum IFN-γ, total immunoglobulin E (IgE), and eosinophils. These results suggest that circulating Foxp3^+^CD4^+ ^cells regulate unknown factor(s) affecting the onset of allergic diseases, which are unrelated to these T_h_1/T_h_2 markers. Measurement of Foxp3^+^CD4^+ ^cells has the potential to aid in evaluating the presence of active inflammation, which cannot be evaluated by known T_h_1- and T_h_2-related markers in patients with allergic diseases [25].

**Figure 3:**
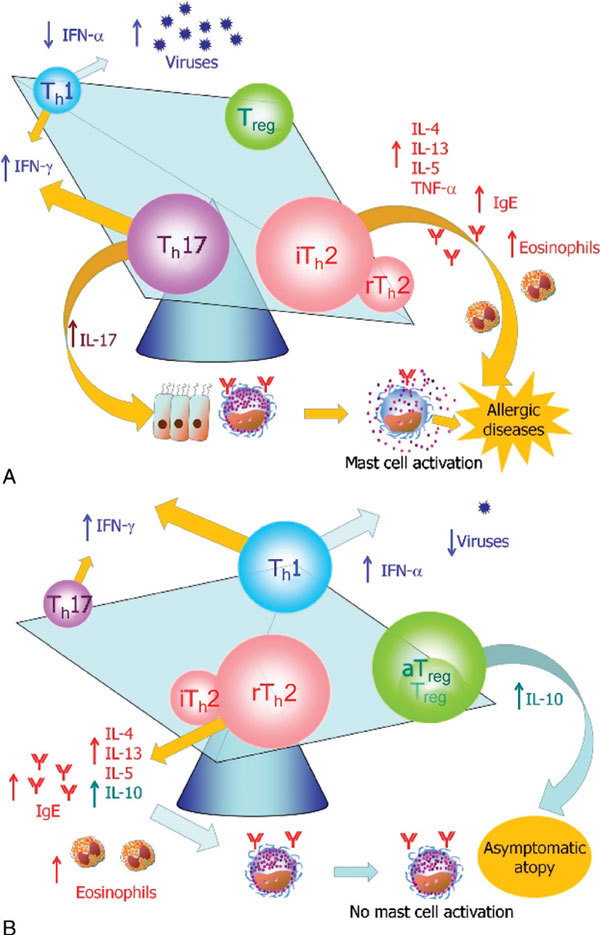
**Onset of allergic diseases may be determined by the ratio of proinflammatory T-cell subsets (T_h_17 and iT_h_2) versus T_reg _subsets**. A, In patients with chronic allergic diseases, proinflammatory T-cell subsets, that is, T_h_17 cells and T_h_2 cells that are capable of producing high levels of TNF-α (iT_h_2 cells) are upregulated. B, In asymptomatic atopic individuals, T_h_2 cells that are capable of producing IL-10 (rT_h_2 cells) may be up-regulated, and T_h_17 cells may be inactivated.

One of the many reasonable explanations for this observation is considered as follows, that is, down-regulation of Foxp3^+ ^aT_reg _cells is often related to up-regulation of T_h_17 cells. In mice, IL-17 activates mast cells to release a proinflammatory cytokine, TNF-α, and thus can cause neutrophilic inflammation [26]. In humans, T_h_17 cells may enhance allergic inflammation by stimulating the tissue resident cells to release TNF-α, and are proven to evoke marked inflammation and airway remodeling. Using the balancing square model, the immunological features of symptomatic allergic diseases may be illustrated as in Figure 3A. Among T_h_2-cell subtypes, TNF-α-rich inflammatory T_h_2 (iT_h_2) cells may be developed by stimulation of dendritic cells with T_h_2 adjuvants associated with allergens or thymic stromal lymphopoietin (TSLP) often found in inflammatory tissues in allergic diseases [27]. Thus, iT_h_2 and T_h_17 cells can be both up-regulated in symptomatic allergic patients, where mast cells are also activated. On the other hand, in asymptomatic controls having similar high levels of IgE and eosinophils, both T_h_2 and aT_reg _cells may be up-regulated. Because of T_h_17 cells, the levels of IFN-γ may be kept at considerably high levels. However, these patients are often infected with viruses at the site of inflammatory tissues because of down-regulation of classic T_h_1 cells [28] capable of producing antiviral cytokine IFN-α.

Among T_h_2 subsets, IL-10-producing T_reg _cells may be up-regulated in asymptomatic atopic individuals. Mast cells are therefore not activated, despite the presence of high levels of IgE antibodies and constant exposure to common allergens as shown in Figure 3B. Nevertheless, it will be necessary to determine why some people do not have marked mast cell activation even though they are sensitized to multiple allergens.

The nT_reg _cells can suppress not only T cells, but also natural killer cells, dendritic cell maturation, and antibody production by B cells [29]. Recently, mast cells and nT_reg_-derived IL-9 are found indispensable in nT_reg_-mediated peripheral tolerance to allograft transplantation in a mouse model [30]. Pollen immunotherapy that is known to induce allergen-specific aT_reg _or Tr1 cells inhibits seasonal increases in IL-9 protein expression and c-Kit^+ ^mast cell infiltration in the nasal mucosa during the pollen season [31]. It is of particular interest to investigate a further relationship between aT_reg _and mast cells in future studies.

## Conclusion

An atopic predisposition is acquired via up-regulation of T_h_2 cells compared with IFN-γ-producing helper T cells (T_h_1 cells and T_h_17 cells) specific for each allergen as shown in Figure 1. Numerous epidemiological studies indicate that microbial components affect the balance between these T-cell types [1]. It should be noted, however, that most of the people who acquired an atopic predisposition in a hygienic environment are still asymptomatic or having very mild symptoms [25]. At present, we have no answer as to why some further develop the clinical manifestations of allergic disease, whereas others remain asymptomatic.

**Figure 4:**
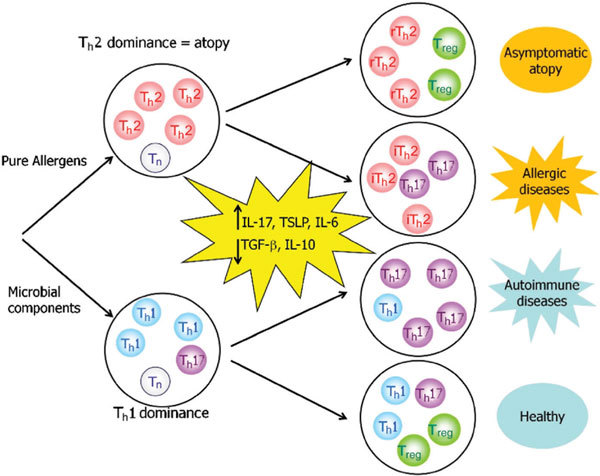
**A model for development of allergic and autoimmune diseases in the absence of microbial components**. Increased ratio of proinflammatory cytokines (IL-17, TSLP, and IL-6) versus regulatory cytokines (IL-10 and TGF-β) may play a key role in determining inflammatory allergic/autoimmune diseases versus asymptomatic individuals.

We have reported that active atopic patients had a lower Foxp3^+^CD4^+ ^ratio than asymptomatic controls having similar levels of serum IFN-γ, total IgE, and eosinophils, [25] suggesting that the development of clinical manifestations of allergic diseases may be determined by the ratio of proinflammatory T-cell subsets (T_h_17 and iT_h_2) versus T_reg _subsets. Increased ratio of proinflammatory cytokines (IL-17, TSLP, and IL-6) versus regulatory cytokines (IL-10 and TGF-β) in severe allergic diseases [8,19,20,27] would activate further the balance shift and form a positive feedback loop in chronic inflammation (Figure 4). Nevertheless, we will have to identify the factors influencing the balance shift of proinflammatory and regulatory T cell population.

## Notes

Supported by the National Institute of Biomedical Innovation Grant ID 05-24.

The authors state that they have no financial relationship with any commercial entity that has an interest in the subject of this article.

## References

[B1] Braun-Fahrländer C Riedler J Herz U Environmental exposure to endotoxin and its relation to asthma in school-age children N Engl J Med 2002 1 869 877 10.1056/NEJMoa020057 12239255

[B2] Mosmann TR Cherwinski H Bond MW Two types of murine helper T cell clone. I. Definition according to profiles of lymphokine activities and secreted proteins J Immunol 1986 1 2348 2357 2419430

[B3] Bach JF The effect of infections on susceptibility to autoimmune and allergic diseases N Engl J Med 2002 1 911 920 10.1056/NEJMra020100 12239261

[B4] Sakaguchi S Ono M Setoguchi R Foxp3^+^CD25^+^CD4^+ ^natural regulatory T cells in dominant self-tolerance and autoimmune disease Immunol Rev 2006 1 8 27 10.1111/j.0105-2896.2006.00427.x 16903903

[B5] Walker MR Carson BD Nepom GT De novo generation of antigen-specific CD4^+^CD25^+ ^regulatory T cells from human CD4^+^CD25^- ^cells Proc Natl Acad Sci USA 2005 1 4103 4108 10.1073/pnas.0407691102 15753318 PMC554797

[B6] Bluestone JA Abbas AK Natural versus adaptive regulatory T cells Nat Rev Immunol 2003 1 253 257 10.1038/nri1032 12658273

[B7] Roncarolo MG Gregori S Battaglia M Interleukin-10-secreting type 1 regulatory T cells in rodents and humans Immunol Rev 2006 1 28 50 10.1111/j.0105-2896.2006.00420.x 16903904

[B8] Bacchetta R Gambineri E Roncarolo MG Role of regulatory T cells and Foxp3 in human diseases J Allergy Clin Immunol 2007 1 227 235 10.1016/j.jaci.2007.06.023 17666212

[B9] Svensson M Maroof A Ato M Kaye PM Stromal cells direct local differentiation of regulatory dendritic cells Immunity 2004 1 805 816 10.1016/j.immuni.2004.10.012 15589169

[B10] Schubert LA Jeffery E Zhang Y Scurfin (Foxp3) acts as a repressor of transcription and regulates T cell activation J Biol Chem 2001 1 37672 37679 10.1074/jbc.M104521200 11483607

[B11] Hori S Nomura T Sakaguchi S Control of regulatory T cell development by the transcription factor Foxp3 Science 2003 1 1057 1061 10.1126/science.1079490 12522256

[B12] Wildin RS Smyk-Pearson S Filipovich AH Clinical and molecular features of the immunodysregulation, polyendocrinopathy, enteropathy, × linked (IPEX) syndrome J Med Genet 2002 1 537 545 10.1136/jmg.39.8.537 12161590 PMC1735203

[B13] Verhagen J Akdis M Traidl-Hoffmann C Absence of T-regulatory cell expression and function in atopic dermatitis skin J Allergy Clin Immunol 2006 1 176 183 10.1016/j.jaci.2005.10.040 16387603

[B14] Allan SE Crome SQ Crellin NK Activation-induced Foxp3 in human T effector cells does not suppress proliferation or cytokine production Int Immunol 2007 1 345 354 10.1093/intimm/dxm014 17329235

[B15] Nakae S Komiyama Y Nambu A Antigen-specific T cell sensitization is impaired in IL-17-deficient mice, causing suppression of allergic cellular and humoral responses Immunity 2002 1 375 387 10.1016/S1074-7613(02)00391-6 12354389

[B16] Steinman L A brief history of T_H17_, the first major revision in the T_H1_/T_H2 _hypothesis of T cell-mediated tissue damage Nat Med 2007 1 139 145 10.1038/nm1551 17290272

[B17] Evans HG Suddason T Jackson I Taams LS Lord GM Optimal induction of T helper 17 cells in humans requires T cell receptor ligation in the context of Toll-like receptor-activated monocytes Proc Natl Acad Sci USA 2007 1 17034 17039 10.1073/pnas.0708426104 17942669 PMC2040448

[B18] Annunziato F Cosmi L Santarlasci V Phenotypic and functional features of human Th17 cells J Exp Med 2007 1 1849 1861 10.1084/jem.20070663 17635957 PMC2118657

[B19] Romagnani S Regulation of the T cell response Clin Exp Allergy 2006 1 1357 1366 10.1111/j.1365-2222.2006.02606.x 17083345

[B20] Schmidt-Weber CB Akdis M Akdis CA TH17 cells in the big picture of immunology J Allergy Clin Immunol 2007 1 247 254 10.1016/j.jaci.2007.06.039 17666214

[B21] Bullens DM Truyen E Coteur L Dilissen E Hellings PW Dupont LJ Ceuppens JL IL-17 mRNA in sputum of asthmatic patients: linking T cell driven inflammation and granulocytic influx? Respir Res 2006 1 135 10.1186/1465-9921-7-135 17083726 PMC1636037

[B22] Linden A Interleukin-17 and airway remodelling Pulm Pharmacol Ther 2006 1 47 50 10.1016/j.pupt.2005.02.004 16286237

[B23] Betts G Twohig J Van den Broek M The impact of regulatory T cells on carcinogen-induced sarcogenesis Br J Cancer 2007 1 1849 1854 10.1038/sj.bjc.6603824 17565340 PMC2359957

[B24] Akdis M Verhagen J Taylor A Immune responses in healthy and allergic individuals are characterized by a fine balance between allergen-specific T regulatory 1 and T helper 2 cells J Exp Med 2004 1 1567 1575 10.1084/jem.20032058 15173208 PMC2211782

[B25] Orihara K Narita M Tobe T Circulating Foxp3^+^CD4^+ ^cell numbers in atopic patients and healthy control subjects J Allergy Clin Immunol 2007 1 960 962 10.1016/j.jaci.2007.05.036 17631953

[B26] Nakae S Suto H Berry GJ Mast cell-derived TNF can promote Th17 cell-dependent neutrophil recruitment in ovalbumin-challenged OTII mice Blood 2007 1 3640 3648 10.1182/blood-2006-09-046128 17197430 PMC1874568

[B27] Liu YJ Thymic stromal lymphopoietin and OX40 ligand pathway in the initiation of dendritic cell-mediated allergic inflammation J Allergy Clin Immunol 2007 1 238 244 10.1016/j.jaci.2007.06.004 17666213

[B28] Gern JE Vrtis R Grindle KA Swenson C Busse WW Relationship of upper and lower airway cytokines to outcome of experimental rhinovirus infection Am J Respir Crit Care Med 2000 1 2226 2231 10.1164/ajrccm.162.6.2003019 11112143

[B29] Miyara M Sakaguchi S Natural regulatory T cells: mechanisms of suppression Trends Mol Med 2007 1 108 116 10.1016/j.molmed.2007.01.003 17257897

[B30] Lu LF Lind EF Gondek DC Mast cells are essential intermediaries in regulatory T-cell tolerance Nature 2006 1 997 1002 10.1038/nature05010 16921386

[B31] Nouri-Aria KT Pilette C Jacobson MR IL-9 and c-Kit+ mast cells in allergic rhinitis during seasonal allergen exposure: effect of immunotherapy J Allergy Clin Immunol 2005 1 73 79 10.1016/j.jaci.2005.03.011 15990777

